# Endodontic remnants are found more than other radiopacities in proposed implant sites

**DOI:** 10.1186/s40729-021-00307-0

**Published:** 2021-04-02

**Authors:** Hamdy A. M. Marzook, Eman A. Yousef, Abeer A. Elgendy

**Affiliations:** 1 Oral and Maxillofacial Surgery Department, Faculty of Dentistry, Mansoura University, 60 Elgomhoria Street, Mansoura, 35516 Egypt; 2 Conservative Dentistry Department, Faculty of Dentistry, Zagazig University, Zagazig, Egypt

**Keywords:** Cone beam computed tomography, Radiopaque lesion, Extraction socket healing, Foreign body, Overfilling, remaining root

## Abstract

**Background:**

Foreign bodies may be a cause of concern in dental implant failure.

**Purpose:**

The aim of the present study was to assess the occurrence and to evaluate the types of radiopacities in dental extraction sites using cone beam computed tomography (CBCT).

**Materials and methods:**

The incidence, location, and types of radiopacities were evaluated in 180 CBCT scans.

**Results:**

Different radiopaque structures could be noted in 84 scans. Foreign bodies and remaining roots were frequently seen. Most of the radiopacities were attributed to remaining endodontic filling in upper and lower jaws in 25 scans in different locations. Remaining roots could be detected in 20 scans. Focal and diffuse radiopaque bony lesions were observed in 16 scans. Tissue response in the form of radiolucency could be seen more with endodontic foreign bodies. Tissue reactions to radiopaque filling remnants were seen in 6.11% of cases.

**Conclusions:**

Foreign body remnants, mostly of endodontic fillings, were frequently seen in CBCT in upper and lower jaws. Evidence of tissue reactions to extraction remnants could be found. Endodontic filling remnants could be seen more in the upper jaw. Thorough examination of implant site for the presence of endodontic foreign body remnants should be stressed. Debridement of the extraction socket should be done carefully in endodontically treated teeth.

## Introduction

Complications related to dental extraction socket healing had been reported. These complications increase the interest into the content and the density of extraction area. A history of periodontal and endodontic pathology may lead to delayed and compromised socket healing [1]. To avoid these complications, total knowledge of the probable causes is recommended. CBCT is an excellent tool to identify metallic foreign objects. It has been proved to be a versatile technique in identifying the foreign objects [2, 3]. Hence, this scanning modality was employed.

Osteoma, hyperplastic calcifications, endodontic fillings, broken tooth fragment, or fractured pieces of fillings and instruments are sometimes visualized in extraction sites [4–8]. These foreign bodies may disturb socket healing. CBCT analysis for extraction sites healing, however, has not been widely evaluated or used in clinical dentistry yet. Therefore, there is a need to establish a well-defined profile for incidence of remnants and the healing of extraction sites in CBCT. The aim of this study was to assess the occurrence of abnormal radiopacities and remnants and to evaluate their effects on the healing of extraction sites using CBCT.

## Materials and methods

The Ethics Committee of the Faculty of Dentistry, Mansoura University, Egypt (Ethical Approval Code Number A13010720), approved this study. All experimental procedures were performed according to approved protocol. The recognized standards have been followed. Extraction sites were studied in 180 CBCT scans for any abnormal radiopacities and their related reactions. Readings from different slices were recorded using Planmeca Romexis Viewer 5.4.1.R. computer program (Planmeca, Italy) for every extraction site. Of the 180 patients, 117 were females and 63 were males. The average age was 34.65 years. Extraction sites with intact implants were excluded. The incidence and location of any abnormal radiopacity in the extraction area were recorded in coronal, sagittal, and axial planes by three investigators. These radiopacities were assigned into their most possible types. The areas adjacent to these radiopacities were studied. Any signs of reactions were recorded. Recorded radiopacities were considered only when there was agreement between two examiners. Impacted teeth in their locations were not considered. Data were collected for the incidence, location, and type of the upper and lower radiopacities in extraction sites and their associated reactions, and evaluated.

## Results

**Fig. 1:**
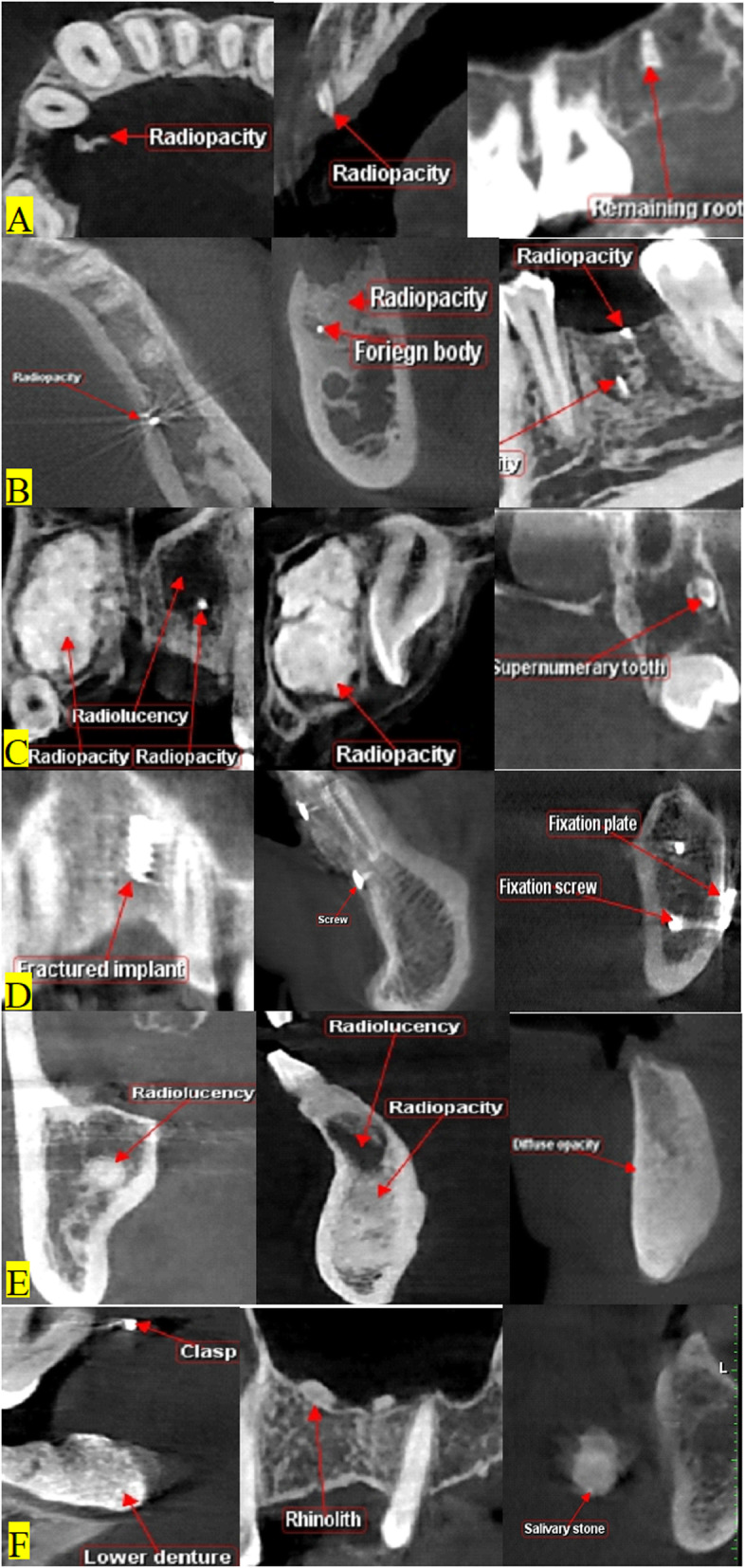
Different forms of radiopacities related to extraction sites are seen in the following forms: **a** fractured piece of bone or remnants of tooth, **b** remnants of endodontic filling with or without bony tissue reactions fragments of bone, **c** pathological lesion, impacted, or supernumerary tooth, **d** fractured implant screw or fixation screws or plates, **e** focal or diffuse bony calcifications or related to resorptive reaction, **f** a nearby clasp of a removable prosthesis, rhinoliths in maxillary sinus, or a salivary gland stone

A total of 84 CBCT scans showed abnormal radiopacities in extraction sites (46.67%). Abnormal radiopacities in extraction sites were recorded from coronal, sagittal, and axial planes at different locations (Table 1). Different forms of radiopacities were seen in upper and lower jaws (Fig. 1). Fractured pieces of bone or remnants of tooth structure were seen in 24 scans (Fig. 1a). Remaining roots could be detected in 20 scans with or without any bony tissue reactions. Remnants of endodontic fillings could be detected in 25 scans (Fig. 1b). Pathological lesion, ectopically impacted or supernumerary tooth were seen in 5 cases (Fig. 1c). Fractured implant screw or fixation screws or plates were found in 4 extraction sites (Fig. 1d). Focal or diffuse bony calcifications were noticed in 16 scans (Fig. 1e) related or un-related to resorptive reaction. A nearby clasp of a removable prosthesis, rhinoliths in maxillary sinus, or a salivary gland stone were seen near extraction sites in 7 scans (Fig. 1f).

**Table 1:** Distribution of radiopacities along the studied 180 CBCT scans

No.	Studied radiopacity	Number of patients	Tissue reactions	% of cases
**1**	**Bony fragments**	**2**	**0**	**1.11%**
**2**	**Pieces of crown**	**2**	**1**	**1.11%**
**3**	**Remaining roots**	**20**	**5**	**11.11%**
**4**	**Pathological lesion, impacted or supernumerary teeth**	**5**	**0**	**2.78%**
**5**	**Fractured implant, or fixation screws or plates**	**4**	**0**	**2.22%**
**6**	**Increased bone density**	**16**	**4**	**8.89%**
**7**	**Removable appliance clasp**	**1**	**0**	**0.56%**
**8**	**Rhinoliths**	**4**	**0**	**2.22%**
**9**	**Salivary gland stone**	**2**	**0**	**1.11%**
**10**	**Brocken instrument**	**1**	**0**	**0.56%**
**11**	**Restorative filling**	**2**	**0**	**1.11%**
**12**	**Endodontic filling remnants**	**25**	**11**	**13.89%**
	**Total**	**84**	**21**	**46.67%**

**Fig. 2:**
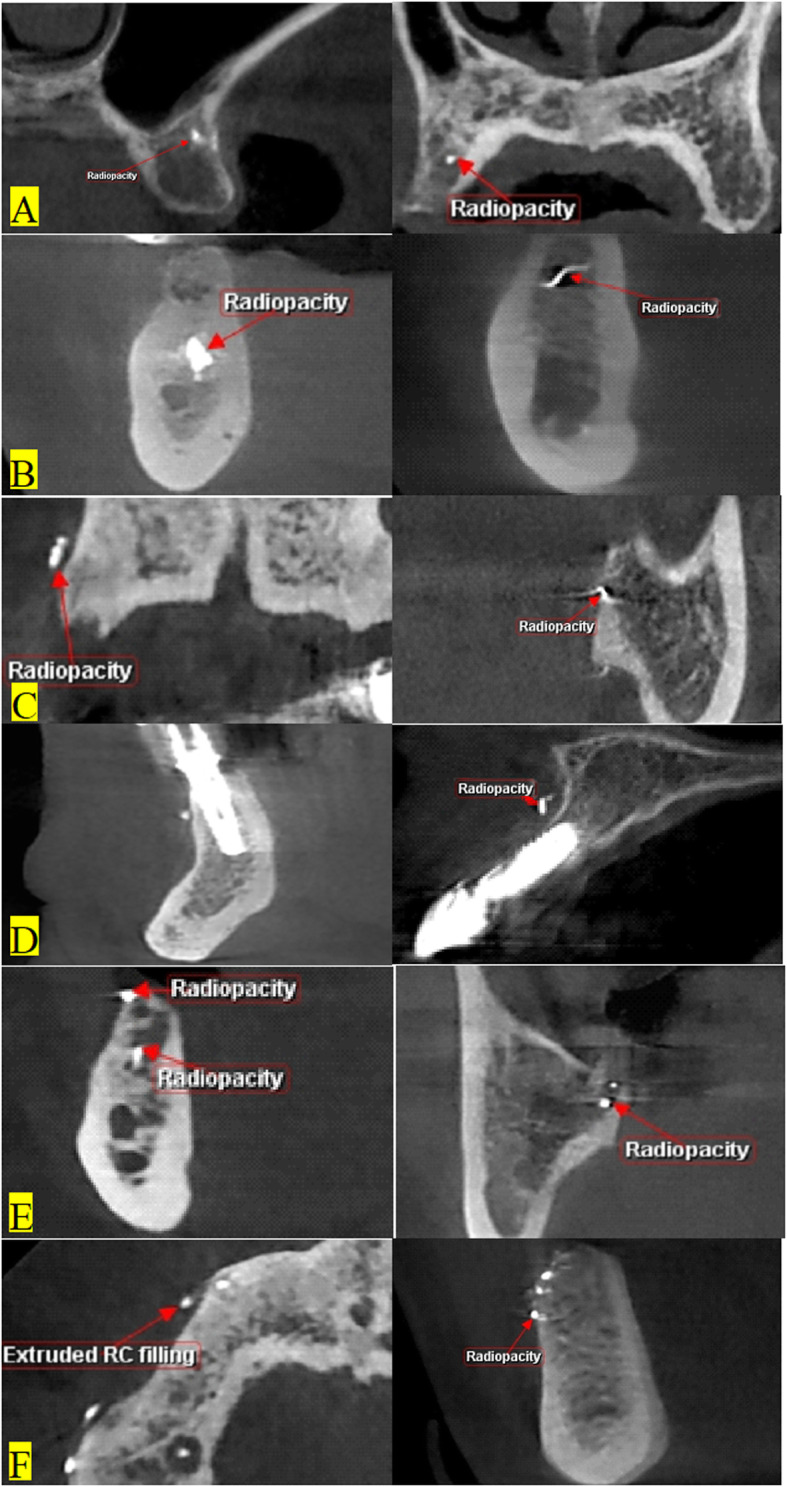
Different forms of remnants of endodontic fillings radiopacities related to extraction sites: **a** small radiopacity deep in maxillary extraction sites, **b** radiopacities in mandibular extraction sites with or without evidence of tissue reaction, **c** remnants of filling radiopacities at the periphery of bone in maxilla and mandible, **d** radiopacities near implant sites in mandible and maxilla, **e** two radiopacities in the same extraction site, **f** more than 2 radiopacities in different sites or in the same extraction site

Radiopaque endodontic foreign bodies were found more in the upper jaw (14) than in the lower jaw (9). Different forms of remnants of endodontic fillings radiopacities were noticed as small radiopacities deep in maxillary extraction sites (Fig. 2a), radiopacities in mandibular extraction sites with or without evidence of tissue reaction (Fig. 2b), remnants of filling radiopacities at the periphery of bone in maxilla and mandible (Fig. 2c), and radiopacities near implant sites in mandible and maxilla (Fig. 2d). More than one radiopacity could be detected. Findings included two radiopacities in the same extraction site (Fig. 2e), and more than 2 radiopacities in different sites or in the same extraction site (Fig. 2f). Different positions of radiopacities in relation to extraction sockets were found. A fractured endodontic file could be detected inside a remaining root fragment.

Tissue response in the form of radiolucency could be seen more (6.11%) with endodontic foreign bodies (Fig. 2b). The incidence of tissue reactions to remaining roots was 2.78%.

We have focused on the presence of radiopaque structures in the extraction sites as an important factor for bone condition. However, this is a pilot study and up till now there are no conclusive data on clinical evaluation of the effect of these structures on implant survival. The authors emphasize that further clinical testing is required. Evidence-based studies have not clearly discussed this before. There is currently lack of available data about the sources.

**Fig. 3:**
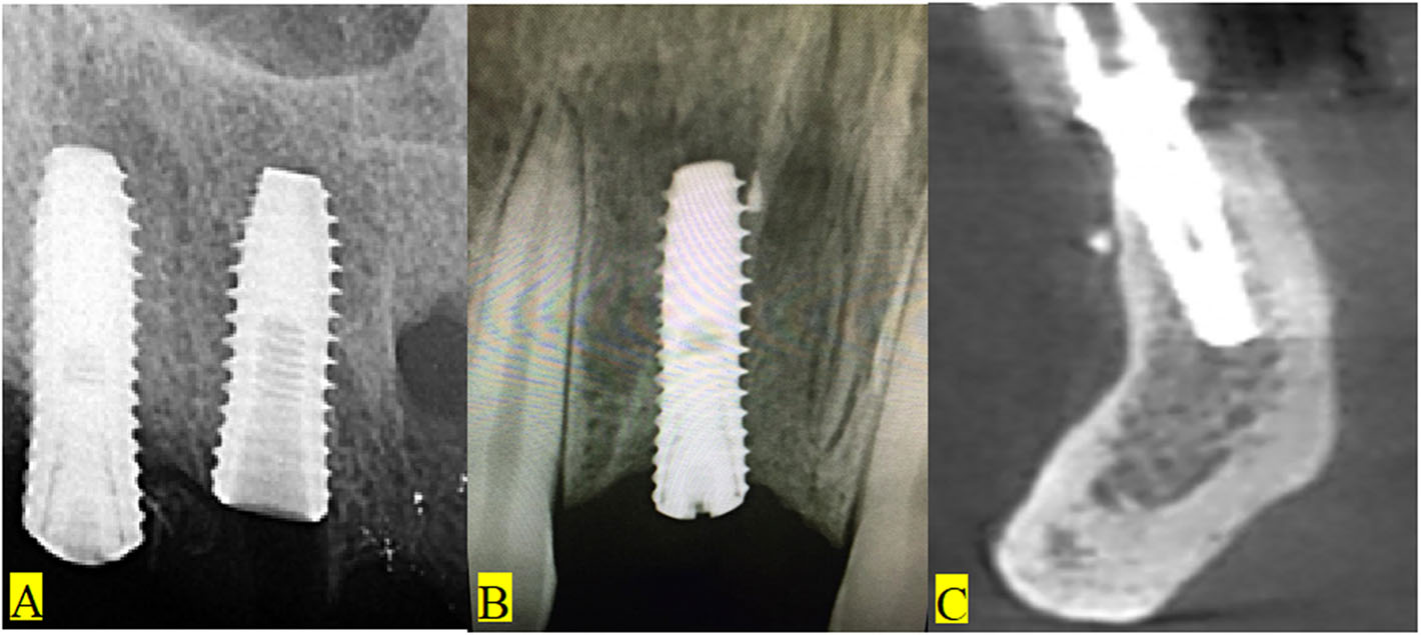
Different radiopaque forms of endodontic filling remnants were frequently seen related to implant fixtures: **a** small radiopacities in maxillary extraction sites away from the implant fixture, **b** radiopacities in close proximity to implant fixture with no evidence of tissue reaction, **c** remnants of filling radiopacities at the periphery of bone near implant fixture

**Fig. 4:**
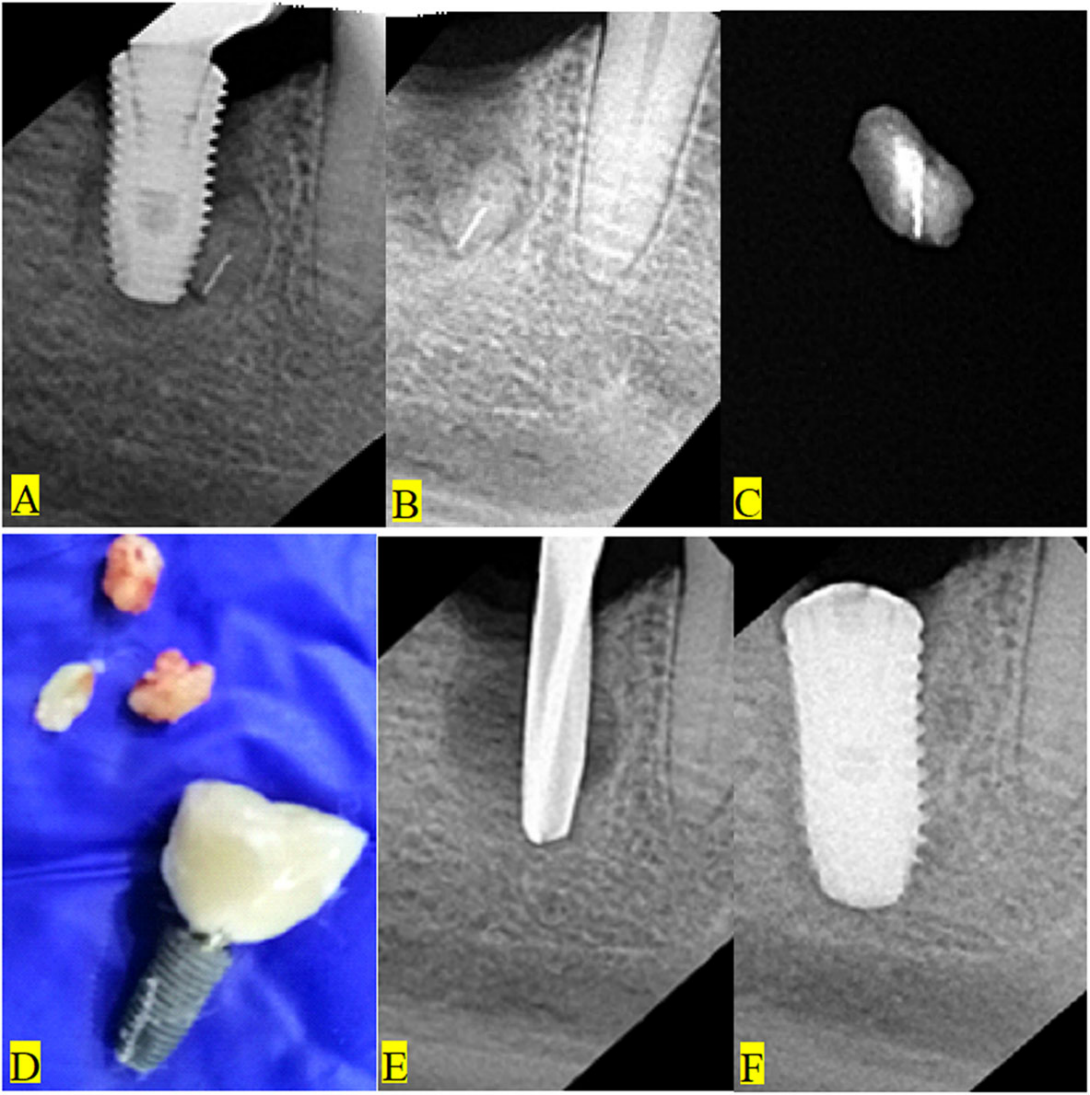
Implant failure due to the presence of a radiopacity of endodontically treated remaining root with a fractured endodontic file. **a** The radiopacity deep in close proximity to the mandibular implant. **b** The radiopacity after removal of the implant with evidence of tissue reaction. **c** Radiographic examination of the removed remaining root with endodontic filling remnants and the broken file. **d** A photograph of the removed implant and the remaining root fragments. **e** Preparation of the site for a longer and wider new implant fixture. **f** The new fixture in place

We are currently conducting a study to assess the success of dental implants placed in relation to similar radiopacities. Different radiopaque remnants were found in relation to implant fixtures (Fig. 3). A case is presented herein about an adult male patient suffering from pain and mobility of an implant supported crown in the lower second molar area. At the time of loading, there were no signs or symptoms of failure. Periapical X-ray examination revealed the presence of a radiopacity adjacent to the failed implant (Fig. 4a). It was diagnosed as implant failure. The plan for care was to remove the implant (Fig. 4b), debride the site (Fig. 4c, d), and to prepare the site for a second screw (Fig. 4e). Remaining root fragments with a fractured piece of endodontic file could be retrieved from the site (Fig. 4c, d). A longer and wider implant screw was inserted (Fig. 4f). Three months later, an abutment was connected to the screw and a porcelain fused to metal crown was cemented to it. The patient was followed up with no complications.

**Fig. 5:**
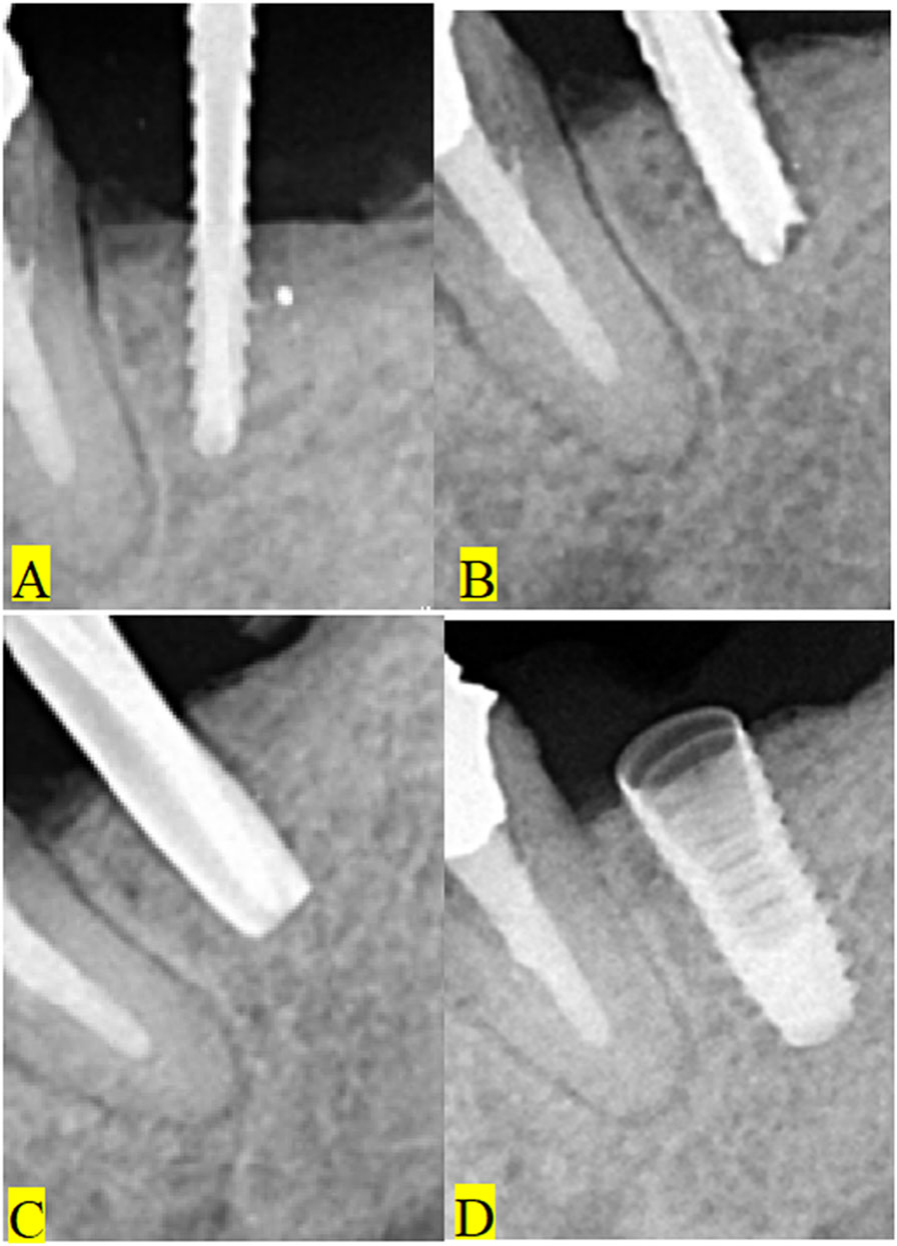
A case showing how to handle the problem of the presence of a radiopacity at the implant site. **a** Initial drilling showing the radiopacity near the small drill. **b** Preparation with a larger drill to include the site of the radiopacity. **c** The final drill is including the radiopacity site. **d** The implant fixture in place with no evidence for the presence of any radiopaque remnants

Another case is presented to show how to handle these problems in the clinics (Fig. 5a–d). CBCT and periapical x-ray examination in an adult male patient revealed the presence of a small radiopacity related to the proposed implant site. A decision was made to include the radiopacity in the healed extraction site of the lower left first molar during the drilling process. Removal of the foreign body was achieved during bone cutting in implant site preparation. The implant fixture was inserted with no signs of abnormal radiopacity (Fig. 5d).

## Discussion

To avoid implant failure, any bony abnormality must be identified. This study was conducted to investigate radiopacities related to extraction sites in CBCT. Reports concerning types of remnants in extraction site are lacking. In this study, remaining root fragments were detected in 11.11% of the studied scans. These results are in agreement with many previous studies [9]. The prevalence of retained root fragments was reported as 11–37%.

Different radiopaque structures related to extraction sites in different forms were noted. Fractured pieces of bone, remnants of tooth structure, remnants of endodontic fillings with or without bony tissue reactions could be detected. Focal or diffuse bony calcifications, a nearby clasp of a removable prosthesis, fractured implant screw or fixation screws or plates were also found. Foreign bodies in the maxillofacial region in general had been studied by many investigators [2, 3]. Instruments breakage during dental extraction was previously presented [2].

An implant failure case was presented. The presence of a remaining root with endodontic filling and a broken file adjacent to the implant screw was the only possible cause of implant failure after loading. This case may suggest the possible role of extraction site remnants in implant failure. Another case was presented to show how to handle this problem in the clinic. Sequential drilling was adjusted to include the radiopaque foreign body in the healed extraction site during bone cutting. Removal of the small radiopaque foreign body was achieved during the drilling process in implant site preparation. The implant fixture was inserted with no evidence for the presence of any radiopaque remnants. Implantologists must give careful attention to the possibility of removal of foreign body radiopacities during implant site preparation.

Radiopaque structures in dental extraction site were put in consideration in this study, due to its possible role in increasing implant failure. The present study did not compare the effect of different sizes and types of calcifications on the implant. There is no evidence that small calcification areas do not affect the implants and hence, further clinical evaluations are required to elucidate this issue.

In this study, the endodontic filling remnants showed higher incidence than previously considered for them. The literature describes many possibilities of endodontic sealer spreading to the periapical region [7, 8]. Extruded sealer can cause problems that vary from mild inflammatory reactions to severe neuro-toxic damage [8]. The findings of the present study proved that there is a high incidence of tissue reactions to foreign bodies in extraction sites. These results coincide with a previous report that implant failures might be related to previous endodontic failures [10].

To prevent implant failures, careful analysis of areas around endodontically compromised teeth was recommended prior to implant placement [11]. The cause of retrograde peri-implantitis is mostly infectious [12]. Remnant bacteria surrounding hard tissues adjacent to implants were found to induce late failures of implants through the development of inflammatory changes [13]. Exposure of implant surface to the bacteria may lead to colonization that potentially trigger pathological features of apical peri-implantitis [13, 14]. It was previously concluded that implant failures seemed to be associated with a history of endodontic failure [10].

Studies investigating tissue reactions to post extraction calcifications are lacking. Tissue reactions might be attributed to the presence of foreign bodies in extraction sites. The periapical sealer extrusion during endodontic treatment may cause the increase and persistence of residual chronic inflammation, which is rarely diagnosed radiographically. Proper attention to the apical limit of root canal obturation appear to be important for the success of endodontic treatment and the peri-radicular tissue repair [15, 16].

Many histological, clinical, and radiographic studies have shown that the apical limit of obturation could affect the final outcome of endodontic treatment [17–20]. Overfills delay the periapical tissue repair process and cause intense inflammatory infiltrate [19]. Even using a biocompatible material as a filling beyond the limit of the apical foramen showed unsatisfactory results [18]. The presence of root canal filling material such as gutta-percha and sealer, as a foreign body, may cause connective tissue responses [21]. Infection and local irritations can be attributed to the presence of overfilled material in soft tissues [22, 23]. It was recommended that overfilling should be avoided [24]. Overfilling induced toxicity may explain the presence of inflammatory reactions in intraosseous implants [25].

This study could be of importance for endodontists, oral surgeons, and implantologists. Endodontist must give careful attention to the working length during obturation and avoid overextension of the filling materials [7, 26–28]. Oral surgeons should thoroughly examine the extraction socket and adjacent tissues after extraction of either endodontically treated teeth or remaining roots. Socket curettage must be performed if endodontic remnants were present. Periapical X-ray at implant placement was recommended to intercept residual infection [29]. The combination of local and systemic risk factors reveals significantly lower implant survival [30]. Localized osteomyelitis secondary to endodontic-implant pathosis was reported [31]. Drilling for implant osteotomy may reactivate bacteria from failed endodontic treatment or residual lesions. Colonization at the implant apex may cause implant failure [29]. Implantologists should examine carefully proposed implant sites for the presence of these radiopaque remnants and consider them during the preparation [32, 33].

## Conclusions

To the best of our knowledge, this study was the first to investigate the radiographic characteristics of calcifications in extraction sites. The results of this study showed that radiopacities are frequently seen in the extraction sites in the upper and lower jaws with wide variations in CBCT. Endodontic filling remnants could be seen more in the upper jaw. More incidence and more tissue reactions were seen with radiopaque remnants of endodontic treatment. These findings should be considered in dental extraction of endodontically treated teeth to prevent possible complications. Immediate postoperative thorough investigation and debridement of the socket is highly recommended.

## Data Availability

The datasets used and/or analyzed during the current study are available from the corresponding author on reasonable request.

## Abbreviations

CBCT: Cone beam computed tomography

## References

[1] Kim J-H, Koo K-T, Capetillo J, Kim J-J, Yoo J-M, Amara HB, Park J-C, Schwarz F, Wikesjö UME (2017). Periodontal and endodontic pathology delays extraction socket healing in a canine model. J Periodontal Implant Sci.

[2] Balaji SM (2013). Burried broken extraction instrument fragment. Ann Maxillofac Surg.

[3] Eggers G, Welzel T, Mukhmadiev D, Wortche R, Hassfeld S, Muhling J (2007). X ray based volumetric imaging of foreign bodies: a comparison of computed tomography and digital volume tomography. J Oral Maxillofac Surg.

[4] Ulutürk H, Yücel E, Akinci HO, Calisan EB, Yildirim B, Gizli A (2019). Multiple calcifying hyperplastic dental follicles. J Stomatol Oral Maxillofac Surg.

[5] Fourcade A, Salmon B, François Le Pelletier F, Anne-Laure Ejeil A-L (2018). Peripheral osteoma of the mandibular crest: a short case study. J Oral Med Oral Surg.

[6] Allareddy V, Vincent SD, Hellstein JW (2012). Incidental findings on cone beam computed tomography images. Int J Dent.

[7] Scolozzi P, Lombardi T, Jaques B (2004). Successful inferior alveolar nerve decompression for dysesthesia following endodontic treatment: report of four cases treated with mandibular sagittal osteotomy. Oral Surg Oral Med Oral Pathol Oral Radiol Endod.

[8] Castro R, Guivarc'h M, Foletti JM, Catherine JH, Guyot L (2018). Endodontic-related inferior alveolar nerve injuries: A review and a therapeutic flow chart. J Stomatol Oral Maxillofac Surg.

[9] Nayyar J, Clarke M, O'Sullivan M, Stassen LF (2015). Fractured root tips during dental extractions and retained root fragments. A clinical dilemma?. Br Dent J.

[10] Chatzopoulos GS, Wolff LF (2017). Implant failure and history of failed endodontic treatment: A retrospective case-control study. J Clin Exp Dent.

[11] Romanos GE, Froum S, Costa-Martins S, Meitner S, Tarnow DP (2011). Implant periapical lesions: etiology and treatment options. J Oral Implantol..

[12] Ramanauskaite A, Juodzbalys G, Tözüm TF (2016). Apical/retrograde periimplantitis/implant periapical lesion: etiology, risk factors, and treatment options: a systematic review. Implant Dent..

[13] López-Martínez F, Gómez Moreno G, Olivares-Ponce P, Eduardo Jaramillo D, Eduardo Maté Sánchez de Val J, Calvo-Guirado JL (2015). Implants failures related to endodontic treatment. An observational retrospective study. Clin Oral Implants Res..

[14] Flanagan D (2016). Implant placement in failed endodontic sites: a review. J Oral Implantol..

[15] Brynolf I (1967). A histological and roentgenological study of the periapical region of human upper incisors. Odontol Revy..

[16] Orstavik D, Mjör IA (1988). Histopathology and x-ray microanalysis of the subcutaneous tissue response to endodontic sealers. J Endod..

[17] Schaeffer MA, White RR, Walton RE (2005). Determining the optimal obturation length: a meta-analysis of literature. J Endod..

[18] Holland R, Mazuqueli L, Souza V, Murata SS, Dezan E, Júnior SP (2007). Influence of the type of vehicle and limit of obturation on apical and periapical tissue response in dogs’ teeth after root canal filling with mineral trioxide aggregate. J Endod..

[19] Suzuki P, Vd S, Holland R, Gomes-Filho JE, Murata SS, Dezan Junior E (2011). Tissue reaction to Endométhasone sealer in root canal fillings short of or beyond the apical foramen. J Appl Oral Sci.

[20] Suzuki P, Vd S, Holland R, Murata SS, Gomes-Filho JE, Dezan Junior E (2010). Tissue reaction of the EndoREZ in root canal fillings short of or beyond an apical foramenlike communication. Oral Surg Oral Med Oral Pathol Oral Radiol Endod.

[21] Fardal O, Johannessen AC, Morken T (2005). Gingivo-mucosal and cutaneous reactions to amalgam fillings. J Clin Periodontol..

[22] Ektefaie MR, David HT, Poh CF (2005). Surgical resolution of chronic tissue irritation caused by extruded endodontic filling material. J Can Dent Assoc..

[23] Muruzábal M, Erasquin J (1966). Response of periapical tissues in the rat molar to root fillings with Diaket and AH-26. Oral Surg Med Oral Pathol.

[24] Holland R, Gomes-Filho JE, Cintra LTA, Queiroz ÍOA, Estrela C (2017). Factors affecting the periapical healing process of endodontically treated teeth. J Appl Oral Sci.

[25] Sousa CJ, Montes CR, Pascon EA, Loyola AM, Versiani MA (2006). Comparison of the intraosseous biocompatibility of AH Plus, EndoREZ, and Epiphany root canal sealers. J Endod..

[26] López-López J, Estrugo-Devesa A, Jané-Salas E, Segura-Egea JJ (2012). Inferior alveolar nerve injury resulting from overextension of an endodontic sealer: non-surgical management using the GABA analogue pregabalin. Int Endod J.

[27] Gatot A, Peist M, Mozes M (1989). Endodontic overextension produced by injected thermoplasticized gutta-percha. J Endod.

[28] Neaverth EJ (1989). Disabling complications following inadvertent overextension of a root canal filling material. J Endod.

[29] Di Murro B, Canullo L, Pompa G, Di Murro C, Papi P. Prevalence and treatment of retrograde peri-implantitis: a retrospective cohort study covering a 20-year period. Clin Oral Investig. 2021. 10.1007/s00784-020-03769-5. 10.1007/s00784-020-03769-5 PMC8310488 33443685

[30] Saridakis SK, Wagner W, Noelken R (2018). Retrospective cohort study of a tapered implant with high primary stability in patients with local and systemic risk factors-7-year data. Int J Implant Dent..

[31] Sussman HI, Moss SS (1993). Localized osteomyelitis secondary to endodontic-implant pathosis. A case report. J Periodontol..

[32] Di Murro B, Papi P, Di Murro C, Pompa G, Gambarini G. Correlation between endodontic pulpal/periapical disease and retrograde peri-implantitis: A case series. Aust Endod J. 2020. 10.1111/aej.12458. 10.1111/aej.12458 33159493

[33] Di Murro B, Pranno N, Raco A, Pistilli R, Pompa G, Papi P (2020). Knowledge and attitude towards retrograde peri-implantitis among Italian implantologists: A cross-sectional survey. Int J Environ Res Public Health.

